# Supplementary material for: Mitochondrial inner membrane permeabilisation enables mtDNA release during apoptosis
Source: EMBO J. 2018 Jul 26;37(17):e99238. doi: 10.15252/embj.201899238 (PMC6120664; doi:10.15252/embj.201899238)
Supplement: Supplementary file 1 — Expanded View Figures PDF [file EMBJ-37-e99238-s001.pdf]

Expanded View Figures

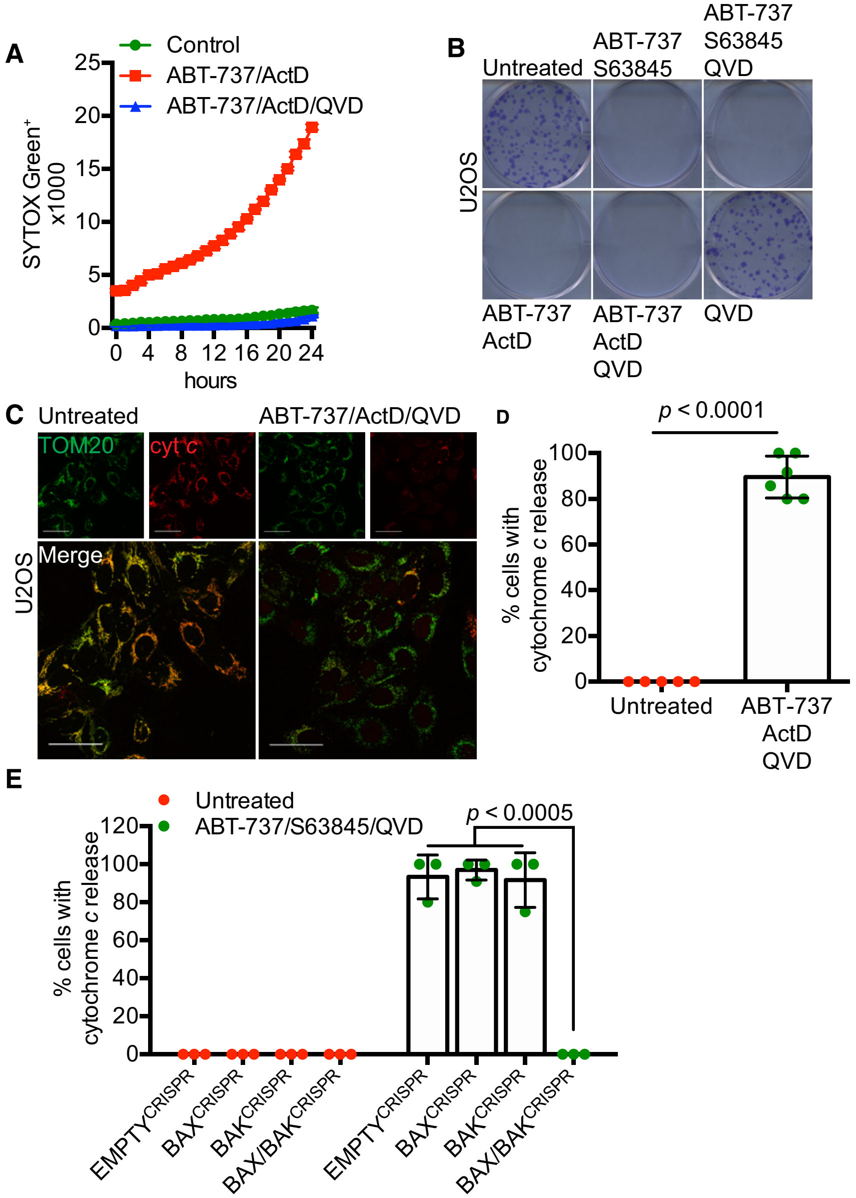

Figure EV1. BAX/BAK-dependent MOMP commits cells to die.

A U2OS cells treated with 10 μM ABT-737 and 1 μM ActD ± 20 μM qVD-OPh. Cell viability was analysed using an IncuCyte live-cell imager and SYTOX Green exclusion. Data are expressed as mean ± SEM, representative of three independent experiments.

B Clonogenic survival assay of U2OS cells treated with 10 μM ABT-737 and 2 μM S63845 ± 20 μM qVD-OPh or 10 μM ABT-737 and 1 μM ActD ± 20 μM qVD-OPh. Representative images from three independent experiments.

C Airyscan images of U2OS cells treated with 10 μM ABT-737, 1 μM ActD and 20 μM qVD-OPh for 3 h, immunostained with anti-TOM20 (green) and anti-cytochrome *c* (red). Scale bar = 10 μm. Representative images from three independent experiments.

D Quantification of cytochrome *c* release from mitochondria. Data are expressed as mean ± SD from three independent experiments and analysed using Student's *t*-test.

E Quantification of cytochrome *c* release from BAX-, BAK-, and BAX/BAK-deleted cells. Data are expressed as mean ± SD from three independent experiments and analysed using Student's *t*-test.

Source data are available online for this figure.

© 2018 The Authors

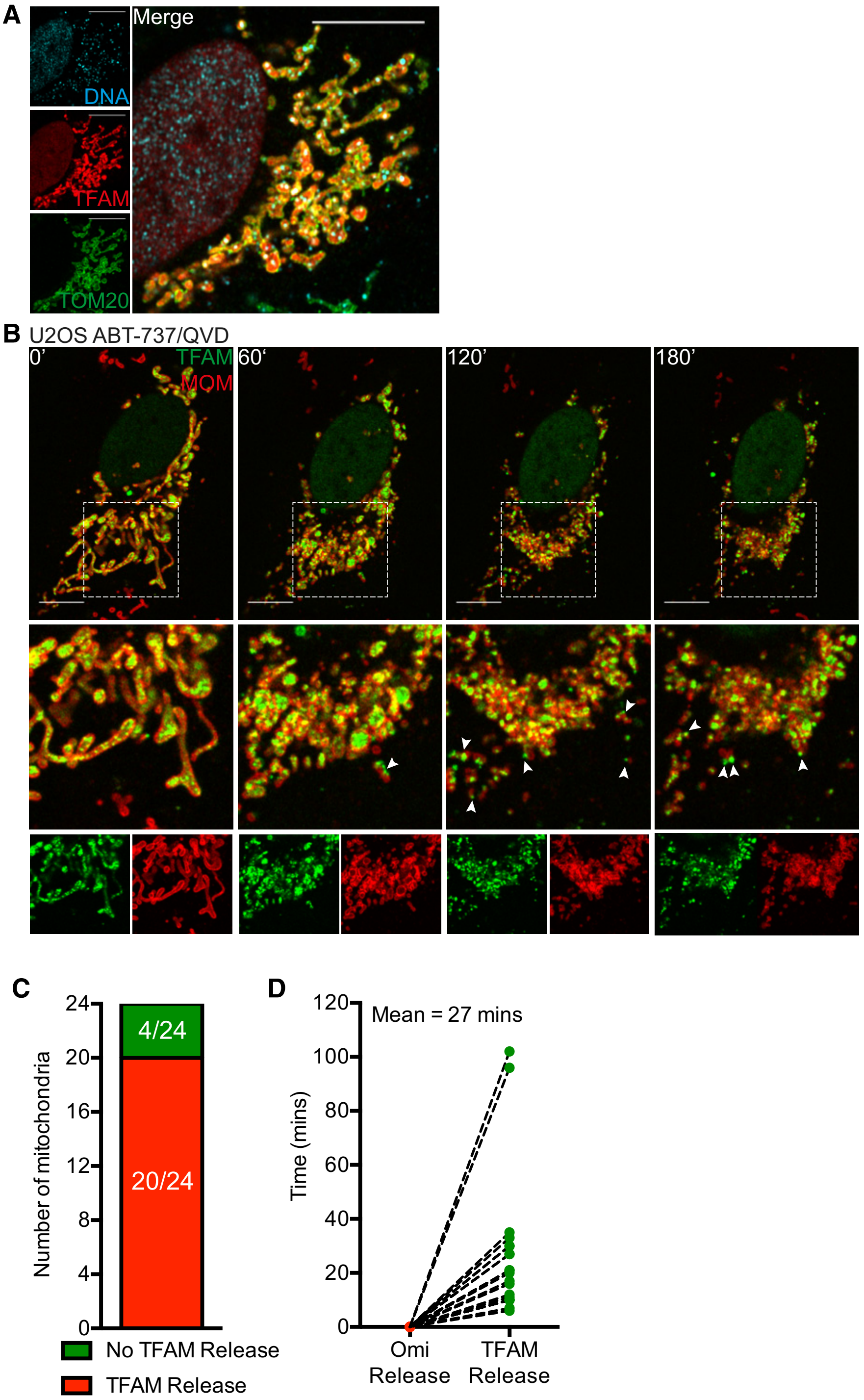

Figure EV2. Kinetic analysis of mitochondrial TFAM release.

A Airyscan image of U2OS cells transfected with TFAM-mScarlet and immunostained with anti-TOM20 and anti-DNA antibodies. Scale bar = 10 µm. Representative images from two independent experiments.

B Live-cell Airyscan imaging of U2OS cells stably expressing JF_{646}-MOM and transiently expressing TFAM-mClover. Cells were treated with 10 µM ABT-737 and 20 µM qVD-OPh at *t* = 0. Scale bar = 10 µm. See Videos EV2 and EV3. Numbers indicate time in minutes. Arrowheads indicate instances of released TFAM.

C Quantification of mitochondria which do or do not release TFAM as assessed by eye from live-cell Airyscan imaging, including Fig 3B and Video EV4. Data are from four cells across two independent experiments.

D Time of Omi release and TFAM released were assessed by eye from live-cell Airyscan imaging, including Fig 3B and Video EV4. Data are from four cells across two independent experiments.

Source data are available online for this figure.

© 2018 The Authors

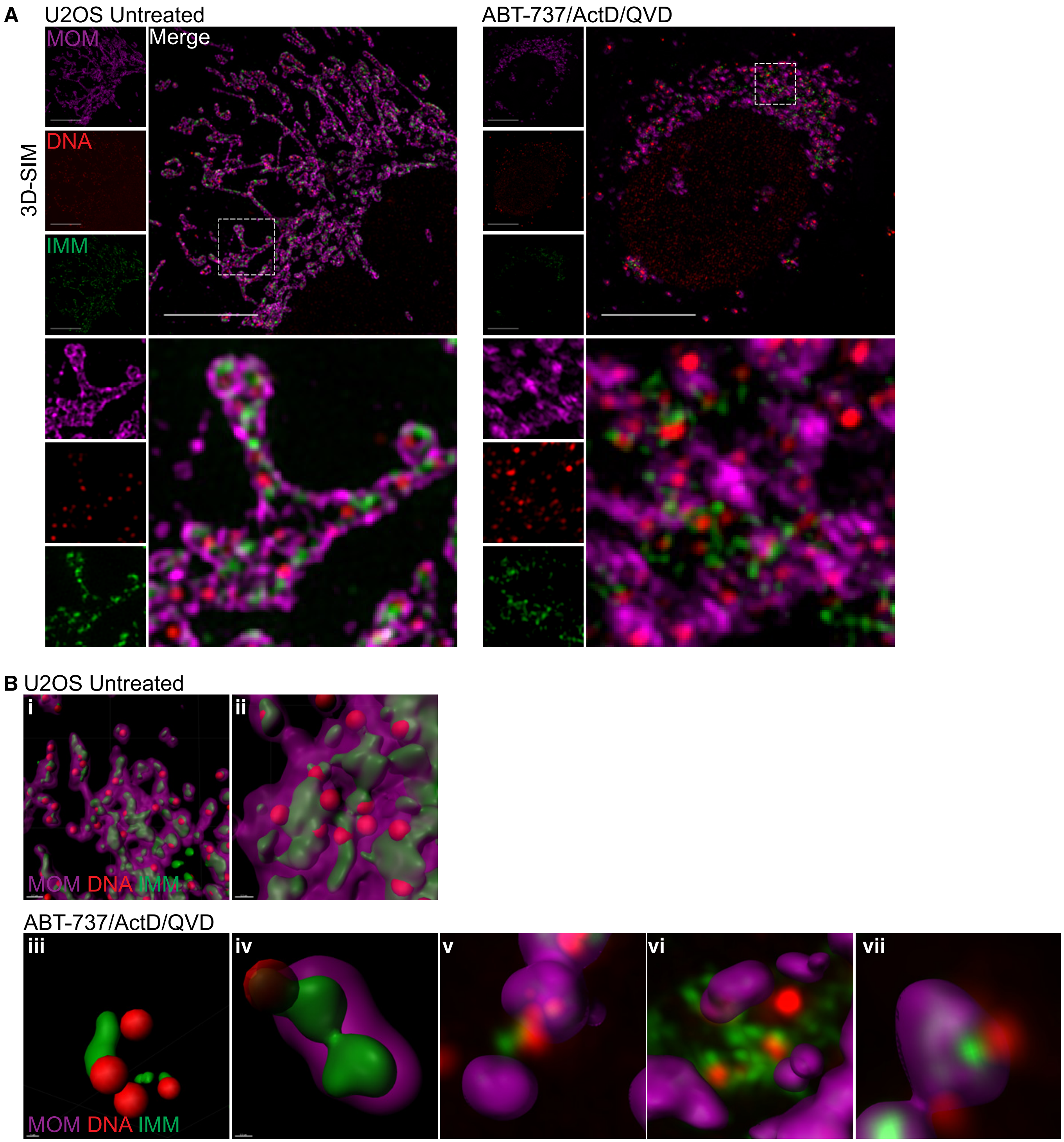

Figure EV3. 3D-SIM analysis of mtDNA release.

A 3D-SIM images of U2OS cells treated with 10 μM ABT-737, 1 μM ActD, and 20 μM qVD-OPh for 3 h. Prior to treatment cells were labelled with JF_{646} (to label SNAP-MOM) and post-fixation were immunostained for IMM (AIF) and DNA. Scale bar = 10 μm. Representative images from three independent experiments.

B Further examples of untreated (i–ii) and treated (iii–iv) U2OS cells 3D-rendered for MOM (magenta), AIF (IMM, green), and DNA (red). (v–vii) show only the MOM 3D-rendered with AIF and DNA in captured form.

Source data are available online for this figure.

© 2018 The Authors

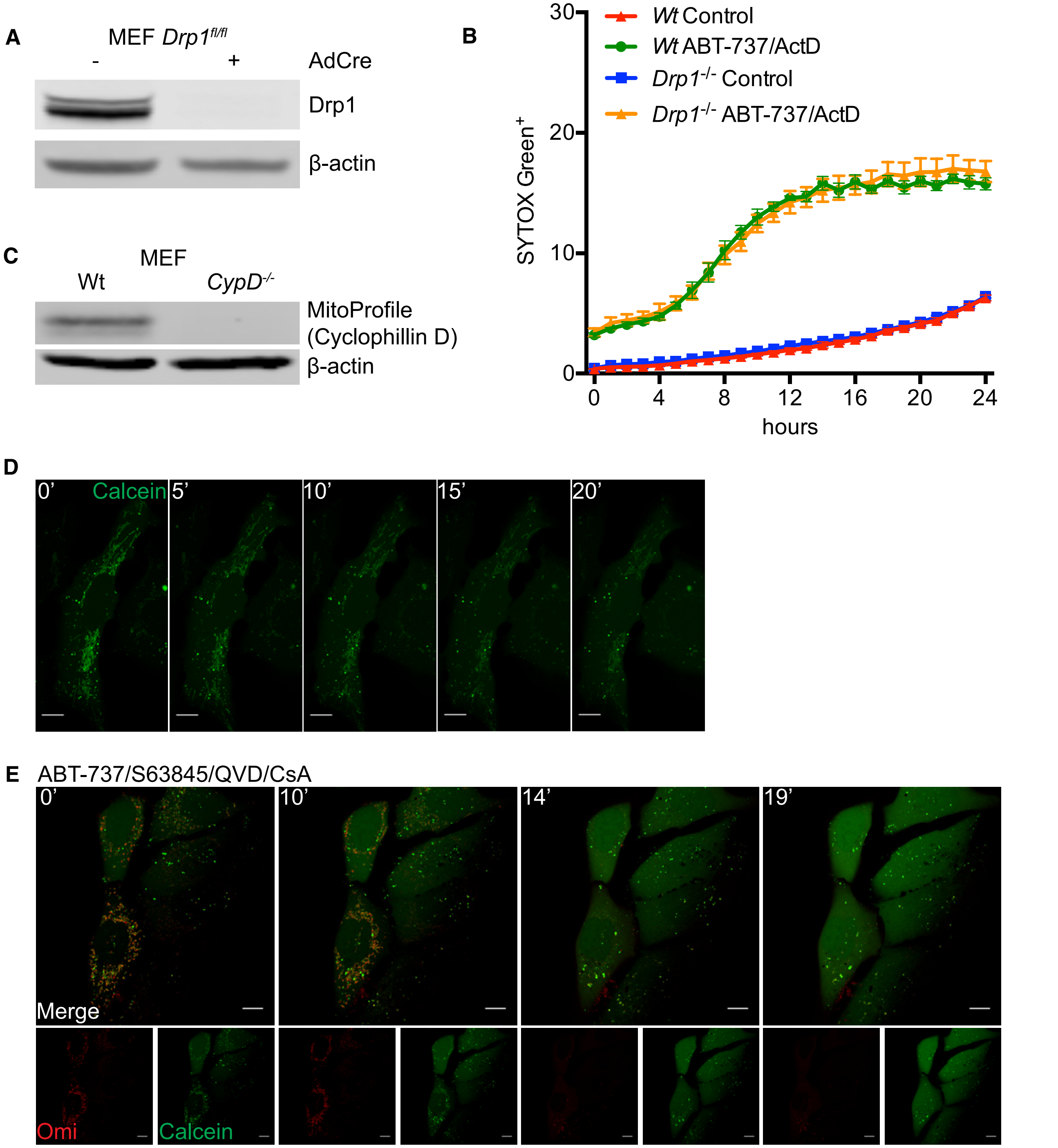

Figure EV4. mtDNA release is independent of mitochondrial fission and the mitochondrial permeability transition pore.

A Expression of Drp1 protein in *Wt* and *Drp1*-deleted MEFs.

B IncuCyte live-imaging of SYTOX Green exclusion in *Wt* and *Drp1*-deleted cells treated with 10 μM ABT-737 and 1 μM ActD to assess cell viability. Data are expressed as mean ± SEM, representative of two independent experiments and have been normalised to starting confluency.

C Expression of CypD protein in *Wt* and *CypD*-deleted MEFs.

D U2OS cells loaded with calcein-AM and $CoCl_2$ and imaged every 30 s for 20 min to show absence of photobleaching. Scale bar = 10 μm. Numbers indicate time in minutes.

E Images from time-lapse live-cell imaging of U2OS cells loaded with calcein-AM and $CoCl_2$ and treated with 10 μM ABT-737, 2 μM S63845 and 20 μM qVD-OPh in the presence of 25 μM CsA at t = 0. Scale bar = 10 μm. See Video EV9. Representative images from two independent experiments. Numbers indicate time in minutes.

 © 2018 The Authors

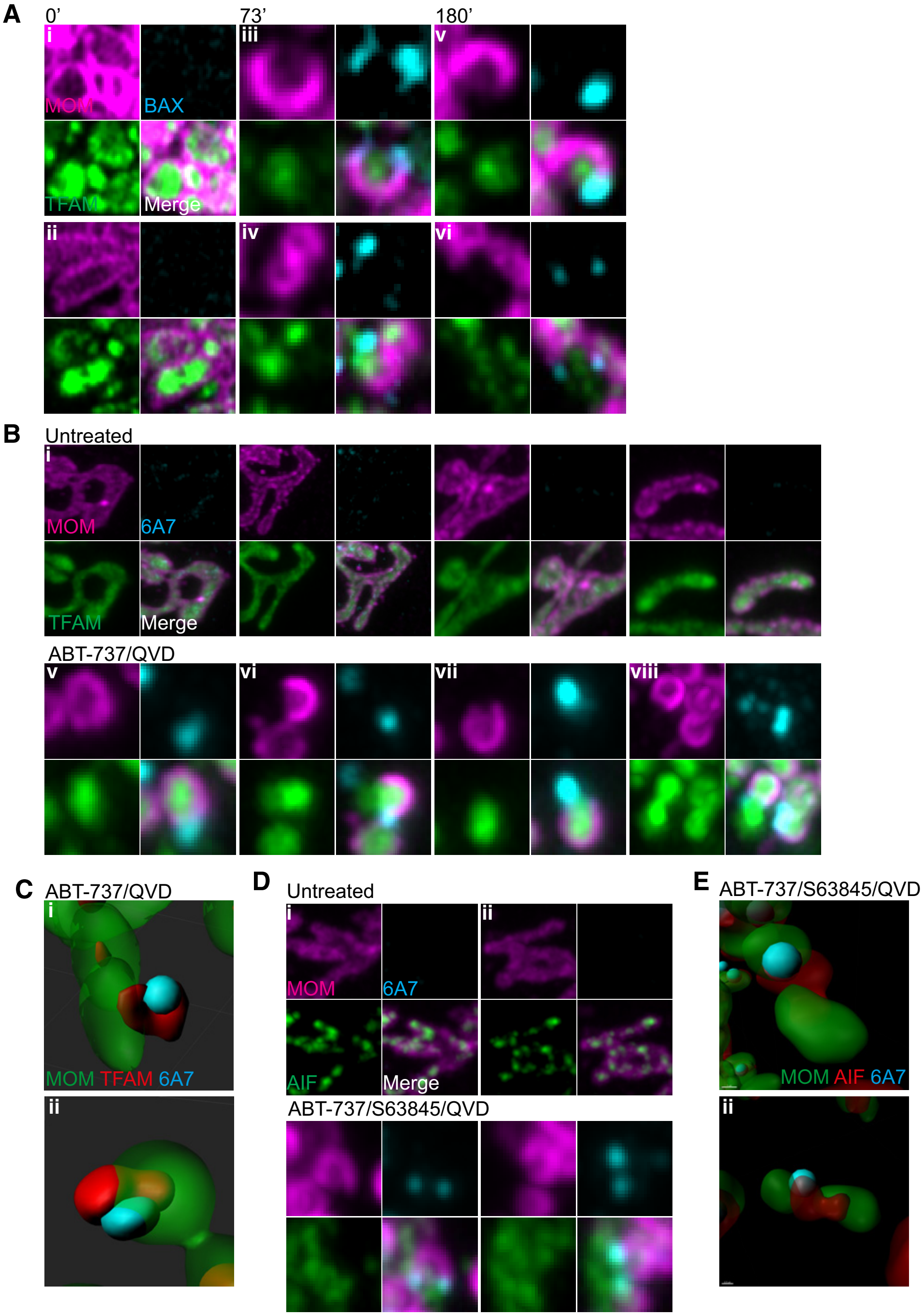

Figure EV5. Mitochondrial inner membrane and mtDNA are extruded through BAX pores on the mitochondrial outer membrane.

A Further examples of stills from live-cell imaging of U2OS *BAX/BAK*^{CRISPR} cells expressing JF_{646}-MOM (magenta) with BAX foci (cyan) and TFAM extrusion (green) from Fig 6A and Video EV10. Cells were treated with 10 μM ABT-737, 2 μM S63845 and 20 μM qVD-OPh at t = 0. Numbers indicate time in minutes.

B Further examples of TFAM extrusion through BAX pores from Fig 6B of U2OS cells stably expressing JF_{646}-MOM (magenta), transiently expressing TFAM-mClover (green) and immunostained for active BAX (6A7, cyan). Cells were treated with 10 μM ABT-737 and 20 μM qVD-OPh for 3 h.

C Further examples of Imaris 3D-renderings from Fig 6B of U2OS cells stably expressing JF_{646}-MOM (green), transiently expressing TFAM-mClover (red) and immunostained for active BAX (6A7, cyan). Cells were treated with 10 μM ABT-737 and 20 μM qVD-OPh for 3 h.

D Further examples of IMM extrusion through BAX pores from Fig 6D of U2OS cells stably expressing JF_{646}-MOM (magenta) and immunostained for AIF (IMM, green) and active BAX (6A7, cyan). Cells were treated with 10 μM ABT-737, 2 μM S63845 and 20 μM qVD-OPh for 3 h.

E Further examples of Imaris 3D-renderings from Fig 6D of U2OS cells stably expressing JF_{646}-MOM (green) and immunostained for AIF (IMM, red) and active BAX (6A7, cyan). Cells were treated with 10 μM ABT-737, 2 μM S63845 and 20 μM qVD-OPh for 3 h.

© 2018 The Authors
