# Supplementary material for: Mitochondrial inner membrane permeabilisation enables mtDNA release during apoptosis
Source: EMBO J. 2018 Jul 26;37(17):e99238. doi: 10.15252/embj.201899238 (PMC6120664; doi:10.15252/embj.201899238)
Supplement: Supplementary file 2 — Video EV1 [file EMBJ-37-e99238-s002.zip › Video1.rtf]

Video 1 – related to Figure 1U2OS cells stably expressing JF646-MOM (magenta) and Omi-mCherry (red) and transiently expressing TFAM-mClover (green) were treated with 10μ ABT-737 and 2μ S62845 in the absence of caspase inhibition. Scale bar = 10μ.
