# Supplementary material for: Mitochondrial inner membrane permeabilisation enables mtDNA release during apoptosis
Source: EMBO J. 2018 Jul 26;37(17):e99238. doi: 10.15252/embj.201899238 (PMC6120664; doi:10.15252/embj.201899238)
Supplement: Supplementary file 4 — Video EV3 [file EMBJ-37-e99238-s004.zip › Video3.rtf]

Video 3 – related to Figure EV Figure 3AZoom of Video 2. Scale bar = 10μ.
