# Supplementary material for: Mitochondrial inner membrane permeabilisation enables mtDNA release during apoptosis
Source: EMBO J. 2018 Jul 26;37(17):e99238. doi: 10.15252/embj.201899238 (PMC6120664; doi:10.15252/embj.201899238)
Supplement: Supplementary file 5 — Video EV4 [file EMBJ-37-e99238-s005.zip › Video4.rtf]

Video 4 – related to Figure 3BU2OS cells stably expressing JF646-MOM (magenta) and Omi-mCherry (red) and transiently expressing TFAM-mClover (green) were treated with 10μ ABT-737, 2μ S62845 and 20μ qVD-OPh. Scale bar as indicated.
