# Supplementary material for: Mitochondrial inner membrane permeabilisation enables mtDNA release during apoptosis
Source: EMBO J. 2018 Jul 26;37(17):e99238. doi: 10.15252/embj.201899238 (PMC6120664; doi:10.15252/embj.201899238)
Supplement: Supplementary file 6 — Video EV5 [file EMBJ-37-e99238-s006.zip › Video5.rtf]

Video 5 – related to Figure 4A3D view of Imaris reconstructions of U2OS cells immunostained for AIF (IMM, red) and DNA (blue). mtDNA outside (blue) and mtDNA inside (green) the IMM signal were quantified. Scale bar = 7μ.
