# Supplementary material for: Mitochondrial inner membrane permeabilisation enables mtDNA release during apoptosis
Source: EMBO J. 2018 Jul 26;37(17):e99238. doi: 10.15252/embj.201899238 (PMC6120664; doi:10.15252/embj.201899238)
Supplement: Supplementary file 7 — Video EV6 [file EMBJ-37-e99238-s007.zip › Video6.rtf]

Video 6 – related to Figure 4A3D view of Imaris reconstructions of U2OS cells treated with 10μ ABT-737, 1μ ActD and 20μ qVD-OPh, immunostained for AIF (IMM, red) and DNA (blue). mtDNA outside (blue) and mtDNA inside (green) the IMM signal were quantified. Scale bar = 7μ.
