# Supplementary material for: Mitochondrial inner membrane permeabilisation enables mtDNA release during apoptosis
Source: EMBO J. 2018 Jul 26;37(17):e99238. doi: 10.15252/embj.201899238 (PMC6120664; doi:10.15252/embj.201899238)
Supplement: Supplementary file 8 — Video EV7 [file EMBJ-37-e99238-s008.zip › Video7.rtf]

Video 7 – related to Figure 4DU2OS cells stably expressing JF646-MOM and transiently expressing TFAM-mScarlet (green) and AIF (1-90)-mClover were treated with 10μ ABT-737, 2μ S62845 and 20μ qVD-OPh. Scale bar = 10μ.
