# Supplementary material for: Mitochondrial inner membrane permeabilisation enables mtDNA release during apoptosis
Source: EMBO J. 2018 Jul 26;37(17):e99238. doi: 10.15252/embj.201899238 (PMC6120664; doi:10.15252/embj.201899238)
Supplement: Supplementary file 9 — Video EV8 [file EMBJ-37-e99238-s009.zip › Video8.rtf]

Video 8 – related to Figure 5FU2OS cells stably expressing JF646-MOM (magenta) and Omi-mCherry (red) loaded with calcein-AM (green) in the presence of CoCl2 were treated with 10μ ABT-737, 2μ S62845 and 20μ qVD-OPh. Scale bar = 10μ.
