# Supplementary material for: Mitochondrial inner membrane permeabilisation enables mtDNA release during apoptosis
Source: EMBO J. 2018 Jul 26;37(17):e99238. doi: 10.15252/embj.201899238 (PMC6120664; doi:10.15252/embj.201899238)
Supplement: Supplementary file 12 — Source Data for Expanded View [file EMBJ-37-e99238-s016.zip › Figure_EV1_Source_Data.pdf]

EV Fig 1A

Hours	Control			ABT-737/ActD			ABT-737/ActD/QVD		
0	0.298	0.39725	0.4635	3.64575	3.74475	3.1815	0.298	0.09925	0.26475
1	0.331	0.46375	0.4965	3.67875	3.8445	3.115	0.29775	0.1325	0.1655
2	0.43025	0.49675	0.4965	4.209	4.3415	3.5795	0.2315	0.1655	0.19875
3	0.49675	0.563	0.596	4.37475	4.905	4.10975	0.26475	0.1655	0.23175
4	0.4635	0.596	0.5295	5.0375	5.2695	4.673	0.298	0.1655	0.29775
5	0.49675	0.629	0.629	5.137	5.5015	4.7725	0.29775	0.1655	0.29775
6	0.49675	0.6955	0.62925	5.36925	6.032	5.336	0.364	0.1655	0.29775
7	0.53	0.7285	0.62925	5.60125	6.1645	5.6345	0.331	0.13225	0.331
8	0.62925	0.6955	0.6625	5.89975	6.6285	5.76675	0.331	0.13225	0.331
9	0.66225	0.6955	0.762	6.1315	6.927	6.26425	0.364	0.13225	0.331
10	0.6955	0.6625	0.76175	6.42975	7.22525	6.72775	0.364	0.13225	0.331
11	0.76175	0.66225	0.9275	6.86075	7.789	7.15925	0.331	0.13225	0.331
12	0.795	0.76175	0.8615	7.4245	8.21975	7.5235	0.397	0.13225	0.364
13	0.86125	0.82825	0.8285	7.789	8.81625	8.15325	0.397	0.16525	0.397
14	0.762	0.86125	0.8285	8.55125	9.31375	8.81625	0.397	0.16525	0.364
15	0.82825	0.828	0.8945	8.98225	9.87725	9.71125	0.43025	0.1985	0.43025
16	0.86125	0.8945	1.027	10.076	10.47375	10.40725	0.43025	0.19875	0.4965
17	0.8945	0.99375	1.1925	11.037	11.236	11.269	0.46325	0.13225	0.563
18	1.027	1.02675	1.3915	11.50125	11.9985	12.297	0.52975	0.265	0.53
19	1.06	1.06025	1.42475	12.86025	12.9595	13.225	0.563	0.265	0.59625
20	1.22625	1.1265	1.6235	13.921	14.18625	13.8215	0.6625	0.298	0.762
21	1.2595	1.16	1.75625	15.1805	14.6835	15.18025	0.72875	0.39725	0.79475
22	1.29225	1.259	2.0545	16.8045	15.87675	16.407	0.86125	0.39725	0.828
23	1.49125	1.29225	2.08775	18.16375	16.77125	17.136	0.99375	0.49675	1.06025
24	1.5575	1.32525	2.18725	19.32375	18.429	19.0585	1.3255	0.8945	1.19275

EV Fig 2D

Untreated	ABT-737/ActD/QVD
0	100
0	80
0	85.71429
0	100
0	80
	91.66666

EV Fig 1E

	Untreated			ABT-737/S63845/QVD		
**EMPTY**^{CRISPR}	0	0	0	100	100	80
**BAX**^{CRISPR}	0	0	0	90.90909	100	100
**BAK**^{CRISPR}	0	0	0	75	100	100
**BAX/BAK**^{CRIS}	0	0	0	0	0	0
