# Supplementary material for: Mitochondrial inner membrane permeabilisation enables mtDNA release during apoptosis
Source: EMBO J. 2018 Jul 26;37(17):e99238. doi: 10.15252/embj.201899238 (PMC6120664; doi:10.15252/embj.201899238)
Supplement: Supplementary file 12 — Source Data for Expanded View [file EMBJ-37-e99238-s016.zip › Figure_EV2_Source_Data.pdf]

**EV Fig 2C**

| | |
|---|---|
| **TFAM Release** | 20 |
| **No TFAM Release** | 4 |

**EV Fig 2D**

| Omi Release | TFAM Release |
|---|---|
| 0 | 21 |
| 0 | 20 |
| 0 | 16 |
| 0 | 10 |
| 0 | 30 |
| 0 | 6 |
| 0 | 10 |
| 0 | 96 |
| 0 | 30 |
| 0 | 10 |
| 0 | 102 |
| 0 | 12 |
| 0 | 21 |
| 0 | 11 |
| 0 | 7 |
| 0 | 17 |
| 0 | 35 |
| 0 | 27 |
| 0 | 27 |
| 0 | 33 |
