# Supplementary material for: Mitochondrial inner membrane permeabilisation enables mtDNA release during apoptosis
Source: EMBO J. 2018 Jul 26;37(17):e99238. doi: 10.15252/embj.201899238 (PMC6120664; doi:10.15252/embj.201899238)

EV Fig 4B

Hours	Wt Control			Wt 10uM ABT-737 + 1uM ActD			$Drp1^{-/-}$ Control			$Drp1^{-/-}$ 10uM ABT-737 + 1uM ActD		
0	0.3834243	0.4288165	0.3741935	3.120244	3.568499	2.960057	0.4401408	0.4291287	0.4864865	2.743363	3.589744	3.913229
1	0.5751364	0.4974271	0.4903226	3.69102	3.896636	3.708987	0.6866197	0.7542263	0.6621622	3.274336	4.536489	4.806465
2	0.5603893	0.5831904	0.5290322	4.185692	4.388844	3.673324	0.8274648	0.8192458	0.7027027	3.539823	4.457594	5.274351
3	0.5603893	0.6689537	0.5935484	4.490107	4.716981	3.887304	0.8802817	0.8322497	0.7567568	3.80531	4.891519	5.359422
4	0.6931131	0.6689537	0.7096774	4.946728	5.127153	4.172611	1.021127	1.027308	0.8783784	3.893805	5.364892	6.167588
5	0.8848252	0.8233276	0.8516129	5.327245	6.234619	5.385164	1.073944	1.092328	0.9324324	4.719764	6.153846	6.635474
6	1.06179	1.012007	1.032258	6.278539	8.28548	6.241084	1.214789	1.274382	1.189189	5.575221	7.179487	7.443641
7	1.209261	1.114923	1.096774	7.267884	9.926169	7.988588	1.373239	1.495449	1.283784	7.256637	8.599606	9.655466
8	1.371479	1.166381	1.264516	9.436834	11.81296	9.486448	1.549296	1.495449	1.445946	8.318584	10.4931	10.76138
9	1.474709	1.200686	1.587097	11.37747	12.83839	10.98431	1.742958	1.703511	1.648649	9.439528	11.55819	11.90983
10	1.636927	1.406518	1.754839	12.82344	14.23298	11.94722	2.007042	1.768531	1.837838	11.06195	13.45168	12.93067
11	1.754903	1.543739	2.012903	13.88889	14.5201	13.19544	2.235915	1.963589	1.972973	11.88791	14.43787	13.78137
12	1.961363	1.698113	2.270968	14.57382	14.88925	14.26534	2.5	2.106632	2.310811	12.65487	15.85799	14.20672
13	2.167822	1.783877	2.387097	14.91629	15.29943	13.87304	2.693662	2.171652	2.567568	13.09735	15.93688	14.92982
14	2.43327	1.921098	2.812903	15.52511	16.8991	15.01427	2.887324	2.561769	2.635135	13.53982	16.80473	15.31263
15	2.669223	2.264151	2.851613	15.37291	16.16079	14.15835	3.045775	2.717815	2.797297	13.59882	17.00197	15.69545
16	3.0379	2.487135	3.019355	15.67732	17.02215	15.04993	3.380282	2.912874	2.878378	14.18879	16.88363	15.90812
17	3.126383	2.813036	3.458065	15.75342	15.50451	14.65763	3.679577	3.276983	3.121622	14.15929	17.357	16.24841
18	3.672025	3.310463	3.780645	16.21004	16.65299	15.12125	3.978873	3.537061	3.472973	14.74926	18.22485	16.71629
19	4.070196	3.464837	3.935484	16.01979	15.99672	14.37232	4.278169	3.862159	3.756757	14.63127	18.38264	16.29094
20	4.497862	3.722127	4.258065	16.62861	16.11977	15.19258	4.542253	4.239272	4.081081	14.57227	18.57988	17.01404
21	4.704321	3.962264	4.580645	16.3242	15.62756	14.90728	4.964789	4.6684	4.459459	14.63127	18.34319	17.26925
22	5.279457	4.802744	5.045161	16.47641	16.65299	15.40656	5.246479	5.20156	5.067567	14.89676	18.61933	17.52446
23	5.677629	5.231561	5.922581	16.70472	16.11977	14.83595	5.721831	5.578674	5.621622	15.19174	17.98816	17.43939
24	6.591948	5.797599	6.464516	16.21004	16.32486	14.76462	6.584507	6.176853	6.5	15.13274	18.10651	17.09911

EV Fig 4A

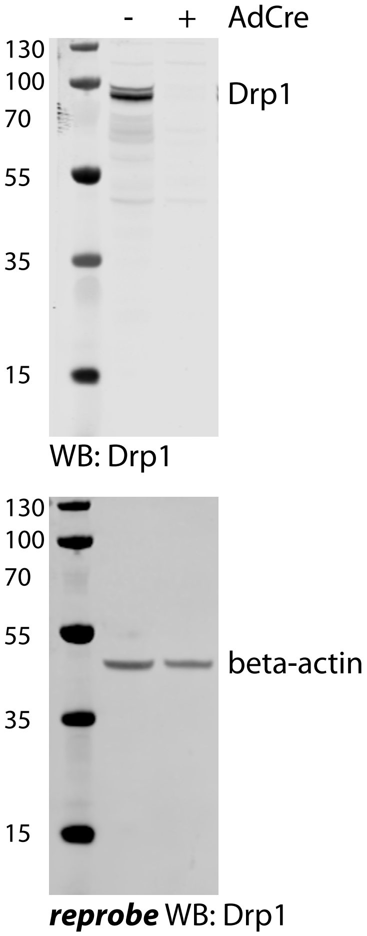

EV Fig 4C

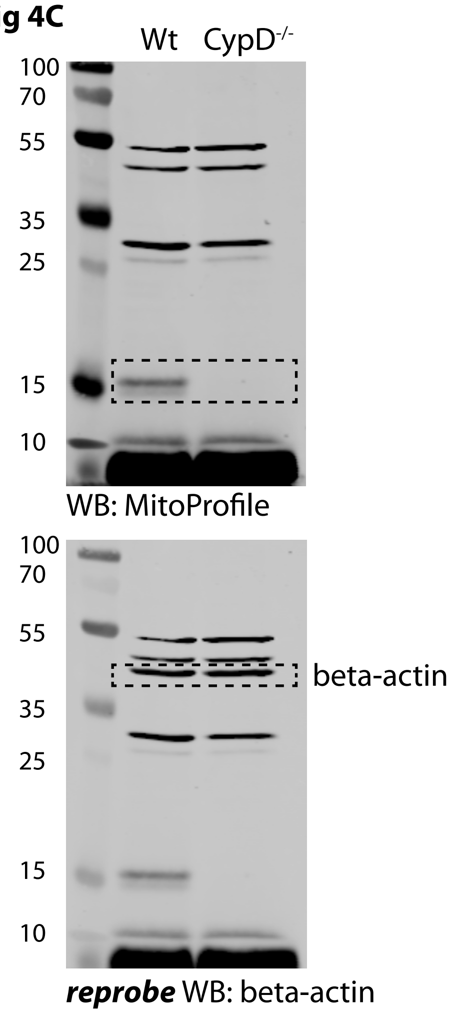

Supplement: Supplementary file 12 — Source Data for Expanded View [file EMBJ-37-e99238-s016.zip › Figure_EV4_Source_Data.pdf]
