# Supplementary material for: Mitochondrial inner membrane permeabilisation enables mtDNA release during apoptosis
Source: EMBO J. 2018 Jul 26;37(17):e99238. doi: 10.15252/embj.201899238 (PMC6120664; doi:10.15252/embj.201899238)
Supplement: Supplementary file 14 — Source Data for Figure 1 [file EMBJ-37-e99238-s012.pdf]

Fig 1C

Untreated	ABT-737/ActD/QVD
0	100
0	100
0	85.71
0	80
0	98.38
0	100
0	100
0	100
	100
	100

Fig 1D

Untreated	ABT-737/ActD/QVD
4.68	83.44
5.33	85.15
4.54	81.81
2.56	73.84
2.85	80.51
3.84	77.87
	80.64

Fig 1G

Hours	U2OS EMPTY^{CRISPR} ABT-737/S63845			U2OS BAX^{CRISPR} ABT-737/S63845			U2OS BAK^{CRISPR} ABT-737/S63845			U2OS BAX/BAK^{CRISPR} ABT-737/S63845		
0	0.1092598	0.1912046	0.05462988	0.1578117	0.0526039	0.1315097	0.1027749	0.05138746	0.03425831	0.09228952	0.184579	0.2461054
1	0.2185195	0.2185195	0.1365747	0.1578117	0.2367175	0.1578117	0.1199041	0.08564577	0.1370332	0.1538159	0.3076317	0.2768686
2	0.1638896	0.464354	0.3004644	0.1578117	0.2630195	0.2630195	0.1712915	0.1712915	0.222679	0.1538159	0.3383949	0.2768686
3	0.2458345	0.7375034	0.7921333	0.4208311	0.5786428	0.5523409	0.4967455	0.445358	0.5652621	0.09228952	0.3691581	0.3076317
4	0.9560229	1.584267	1.693526	0.999474	0.973172	0.8942662	0.9421034	0.890716	1.233299	0.06152635	0.3999212	0.3383949
5	1.693526	2.48566	2.594919	1.735928	1.709627	1.946344	1.490236	1.524495	1.901336	0.09228952	0.3691581	0.3691581
6	2.594919	3.933352	3.550942	3.077328	2.446081	3.051026	2.380952	2.500856	2.963344	0.09228952	0.3383949	0.3999212
7	3.796777	5.408359	5.025949	4.103104	3.366649	4.208312	3.117506	3.374443	3.768414	0.09228952	0.3691581	0.4922107
8	4.53428	6.364381	6.255122	5.576013	4.418727	5.234087	3.871189	4.213772	4.710517	0.1230527	0.4614476	0.5845003
9	5.244469	7.265774	6.96531	7.02262	5.339295	6.128354	4.65913	5.121617	5.618362	0.1230527	0.4922107	0.5845003
10	5.599563	8.740781	7.975963	8.311415	6.338769	6.89111	5.361425	5.823912	6.594724	0.2461054	0.522974	0.6460266
11	6.364381	9.724119	8.713467	9.521304	7.15413	7.916886	6.08085	6.611854	7.69099	0.2768686	0.6460266	0.7690793
12	6.774106	10.73477	9.478285	10.25776	7.995792	8.78485	6.491949	7.416924	8.376156	0.3383949	0.6152635	0.7998425
13	7.293089	11.60885	9.942638	11.12572	8.521831	9.337191	7.108599	8.067831	9.164097	0.4614476	0.6152635	0.7998425
14	7.648183	12.72876	10.35236	11.57286	9.284587	9.416097	7.69099	8.633094	9.883522	0.522974	0.6152635	0.7998425
15	8.358372	13.05654	10.70746	12.1515	9.73172	9.889532	8.10209	9.078451	10.62008	0.4922107	0.5845003	0.8306057
16	8.549577	13.82136	11.11718	12.70384	10.15255	10.25776	8.547448	9.609455	11.28811	0.522974	0.6460266	0.892132
17	9.01393	14.36766	11.49959	13.01946	10.96791	10.59968	8.958548	9.934909	11.76773	0.4922107	0.6767898	0.8306057
18	9.068561	14.85933	11.99126	13.30878	11.46765	10.88901	9.318259	10.38027	12.19596	0.6152635	0.707553	0.8306057
19	9.560229	15.21442	12.23709	13.65071	11.80957	11.36244	9.472422	10.70572	12.35012	0.6460266	0.707553	0.9228952
20	9.806064	15.76072	12.45561	14.01894	11.86218	11.59916	9.746489	11.08256	12.72696	0.707553	0.6767898	0.9228952
21	9.915323	15.97924	12.72876	14.17675	12.04629	11.88848	9.832134	11.16821	13.01816	0.7998425	0.7383162	0.9228952
22	10.16116	16.17044	12.86534	14.49237	12.33561	12.04629	10.19185	11.4594	13.27509	0.8613688	0.7690793	0.8613688
23	10.5982	16.27971	13.05654	14.8343	12.91426	12.2041	10.1062	11.6307	13.58342	0.9228952	0.707553	0.9844216
24	10.7894	16.60748	13.38432	15.01841	12.96686	12.49342	10.32888	11.81912	13.73758	0.9228952	0.8306057	0.9844216

Fig 1I

EMPTY^{CRISPR}	*BAX* ^{CRISPR}	*BAK* ^{CRISPR}	*BAX/BAK* ^{CRISPR}
72.08	83.33	83.15	5.29
71.3	85.71	80.77	6.37
77.14	89.13	88.24	13.9
76.47	88.19	93.12	15.32
92.59	89.02	89.97	13.71
79.34	81.19	92.89	6.8
	72.53	94.85	9.05

Fig 1F

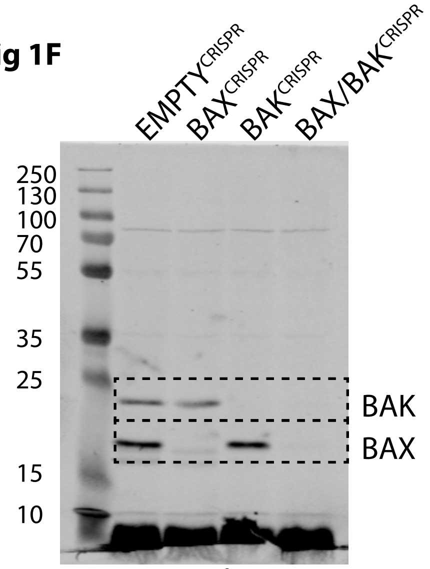

WB: BAX and BAK

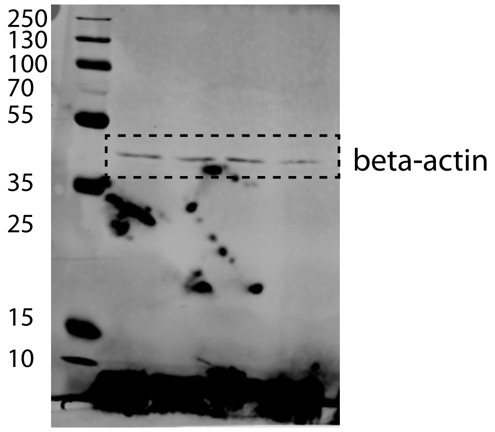

reprobe WB: beta-actin
