# Supplementary material for: Mitochondrial inner membrane permeabilisation enables mtDNA release during apoptosis
Source: EMBO J. 2018 Jul 26;37(17):e99238. doi: 10.15252/embj.201899238 (PMC6120664; doi:10.15252/embj.201899238)
Supplement: Supplementary file 15 — Source Data for Figure 2 [file EMBJ-37-e99238-s013.pdf]

Fig 2A

Hours	Control			QVD			ABT-737/S63845			ABT-737/S63845/QVD		
0	0	0.0795	0.099375	0.059625	0.09605	0.086125	0.07285	0.033075	0.059625	0.069525	0.039725	0.096075
1	0.13915	0.13585	0.10935	0.099375	0.1226	0.089425	0.1027	0.06625	0.099375	0.109325	0.046325	0.106025
2	0.125875	0.139175	0.13915	0.1027	0.115975	0.099375	0.437475	0.285	0.3281	0.125925	0.0596	0.0994
3	0.152425	0.152425	0.16235	0.122575	0.135875	0.106	1.766625	1.402025	1.40205	0.169	0.159075	0.165675
4	0.15245	0.15575	0.169	0.115975	0.14245	0.132525	2.790875	2.108025	1.9125	0.222025	0.222025	0.25185
5	0.165675	0.159075	0.159075	0.1193	0.15905	0.13585	3.450475	2.446125	2.615175	0.2618	0.338025	0.324775
6	0.1889	0.165675	0.178925	0.13585	0.1723	0.122575	3.7985	2.837275	3.0925	0.3745	0.4242	0.377825
7	0.1922	0.159075	0.19885	0.152425	0.19885	0.1292	4.1101	3.032825	3.344425	0.500475	0.536925	0.510425
8	0.2154	0.182275	0.208775	0.185575	0.205475	0.17565	4.39845	3.26815	3.493575	0.609825	0.642975	0.652925
9	0.218725	0.175625	0.231975	0.1756	0.19885	0.175625	4.4084	3.31125	3.533325	0.702675	0.782225	0.79215
10	0.238575	0.205475	0.22865	0.185575	0.218725	0.1657	4.60725	3.301325	3.6394	0.8452	0.875	0.911475
11	0.2618	0.195525	0.208775	0.168975	0.22205	0.172325	4.729875	3.55985	3.702375	0.9844	1.09045	1.0971
12	0.255175	0.24525	0.2585	0.165675	0.2585	0.17895	4.8923	3.55985	3.841625	1.126925	1.252875	1.186575
13	0.255175	0.2353	0.285025	0.17895	0.261775	0.2187	5.071325	3.619525	3.798475	1.329125	1.4683	1.3954
14	0.251875	0.258525	0.251875	0.1723	0.2817	0.258475	5.05475	3.662625	3.8615	1.511425	1.58435	1.567775
15	0.265125	0.281725	0.28835	0.17895	0.285025	0.278375	5.164125	3.7256	3.94435	1.710325	1.783225	1.70035
16	0.285	0.314825	0.291625	0.195525	0.3082	0.331425	5.306675	3.675875	3.924475	1.899225	1.94895	1.829625
17	0.29495	0.301575	0.291625	0.202175	0.33145	0.3248	5.329875	3.7057	4.0637	2.0517	2.064975	1.98875
18	0.334725	0.3546	0.298275	0.225325	0.328075	0.377825	5.30005	3.646025	4.1565	2.19755	2.3003	2.17435
19	0.29165	0.344675	0.3281	0.24855	0.3712	0.38115	5.1774	3.709025	4.047075	2.2539	2.456075	2.24725
20	0.344675	0.354625	0.3513	0.27505	0.407625	0.45405	5.246975	3.6626	4.1565	2.426225	2.58205	2.316875
21	0.324825	0.43085	0.3546	0.27175	0.417575	0.38445	5.187325	3.64605	4.10675	2.4826	2.668225	2.363275
22	0.3546	0.3712	0.354625	0.3314	0.5137	0.4176	5.28675	3.609575	4.086875	2.635075	2.847225	2.4826
23	0.348	0.410975	0.41095	0.31815	0.4938	0.410975	5.19065	3.60295	4.03715	2.7345	2.88365	2.5754
24	0.3745	0.500475	0.454025	0.367875	0.6032	0.517025	4.53105	3.75875	4.33215	2.98975	3.201875	2.8671

Fig 2C

Ifnb1			*Irf7*			*Oasl1*		
0h	3h	8h	0h	3h	8h	0h	3h	8h
0.99	167.87	127.33	1.27	1.98	1.75	1.95	13.99	15.27
1.04	190.13	123.83	0.87	1.6	1.97	0.71	14.99	17.95
0.98	180.34	95.04	0.9	2.13	1.77	0.72	17.18	16.41

Fig 2D

Ifnb1		
Untreated	2h -QVD	2h +QVD
1.36	3.73	73.42
0.47	4.23	83.32
1.56	4.48	73.02

Fig 2F

	Ifnb1					
	Untreated			ABT-737/S63845/QVD		
**EMPTY^{CRISPR}**	0.544771	1.164145	1.576809	185.1186	195.7593	213.5612
**STING$^{CRISPR\ \#1}$**	0.776829	1.183569	1.08763	13.67806	12.39962	10.3782
**STING$^{CRISPR\ \#2}$**	1.053269	0.976844	0.971931	7.650145	7.219899	6.943666
**STING$^{CRISPR\ \#3}$**	1.220512	0.892007	0.918522	41.71678	41.51345	40.11172

Fig 2H

	Ifnb1					
	Untreated			ABT-737/S63845/QVD		
**EMPTY^{CRISPR}**	0.865441	0.998849	1.156812	86.83372	99.04528	110.7511
**BAX^{CRISPR}**	0.883305	0.991826	1.141442	71.07622	81.69583	86.45236
**BAK^{CRISPR}**	0.774513	0.99639	1.295812	81.80103	70.32348	82.6251
**BAX/BAK^{CRISPR}**	1.004557	0.970778	1.025429	23.48593	25.61202	24.79303

Fig 2E

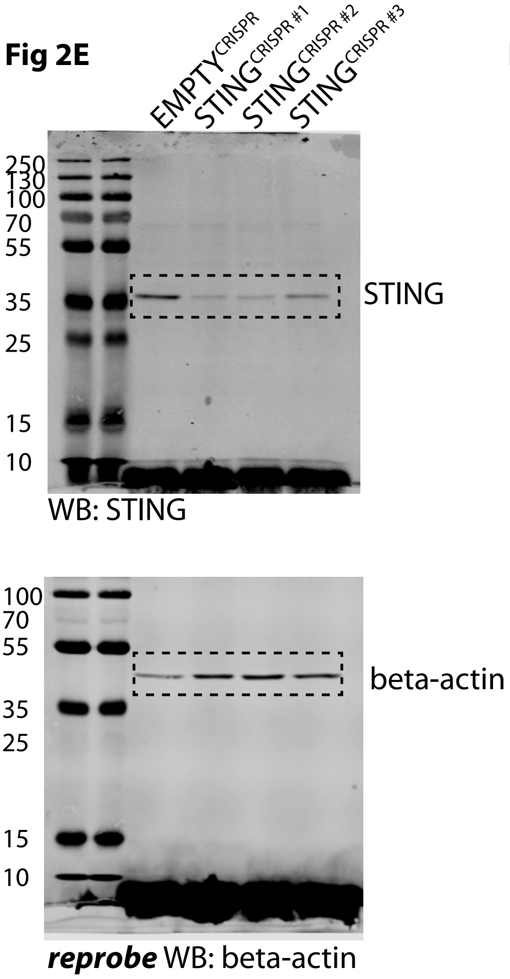
EMPTY^CRISPR
STING^CRISPR #1
STING^CRISPR #2
STING^CRISPR #3
250
130
100
70
55
35
25
15
10
STING
WB: STING
100
70
55
35
25
15
10
beta-actin
reprobe WB: beta-actin

Fig 2G

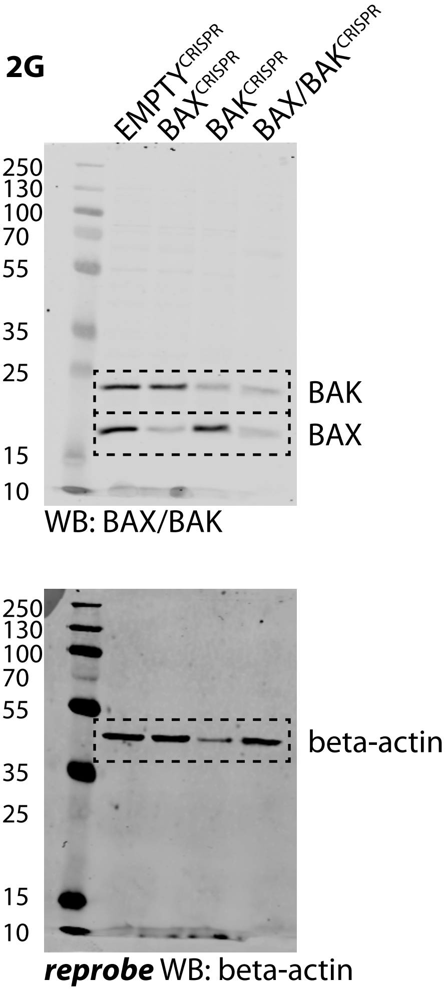
EMPTY^CRISPR
BAX^CRISPR
BAK^CRISPR
BAX/BAK^CRISPR
250
130
100
70
55
35
25
15
10
BAK
BAX
WB: BAX/BAK
250
130
100
70
55
35
25
15
10
beta-actin
reprobe WB: beta-actin
