# Supplementary material for: Mitochondrial inner membrane permeabilisation enables mtDNA release during apoptosis
Source: EMBO J. 2018 Jul 26;37(17):e99238. doi: 10.15252/embj.201899238 (PMC6120664; doi:10.15252/embj.201899238)
Supplement: Supplementary file 16 — Source Data for Figure 3 [file EMBJ-37-e99238-s014.pdf]

Fig 3D

Mins	Pore Size			Omi Release		
0	0	0	0	1	1	1
1	0	0	0	0.922689	0.977456	1.053168
2	0	0	0	0.843128	0.907561	0.927209
3	0	0	0	0.956277	0.949354	0.958964
4	0	0	0	0.858217	0.8774	0.932668
5	0	0	0	0.877579	1.085285	0.804707
6	0	0	0	0.920894	0.951773	0.864062
7	0	0	0	0.834129	0.924391	0.871038
8	0	0	0	0.823561	0.842658	0.821904
9	0	0	0	0.706118	0.706367	0.861272
10	0	0	0	0.619921	0.822585	0.828304
11	0	0	0	0.757181	0.953575	0.7553
12	0	0	0	0.918939	0.953163	0.751145
13	0	0	0	0.832924	0.931803	0.764005
14	0	0	0	0.644214	0.878995	0.838889
15	0	0	0	0.732479	1.051212	0.802372
16	0	0	0	0.437165	0.953111	0.804252
17	0	0	0	0.429552	0.840393	0.810257
18	0	0	0	0.230025	0.669	0.77465
19	0	0	0	0.168189	0.331978	0.765855
20	0	0	0	0.140055	0.284832	0.705195
21	0	0	0	0.108127	0.23259	0.499348
22	0	0	0	0.102491	0.211642	0.410391
23	0	0	0	0.095355	0.202584	0.250463
24	0	0	0	0.091287	0.196047	0.250463
25	0	0	0	0.09381	0.200165	0.23278
26	0.171	0.242	0	0.096537	0.190694	0.176943
27	0.259	0.57	0.245	0.090696	0.191312	0.139668
28	0.494	0.537	0.484	0.091355	0.216223	0.130205
29	0.815	1.316	0.57	0.091378	0.189459	0.127051
30	0.663	1.043	0.843	0.086992	0.186319	0.13154
31	0.79	1.212	0.831	0.088583	0.189408	0.123563
32	0.972	1.273	1.035	0.09081	0.181584	0.124534
33	1.028	1.266	0.929	0.085288	0.184569	0.121956
34	1.002	1.428	0.948	0.086288	0.184055	0.11777
35	0.955	1.398	1.099	0.090696	0.18493	0.120379
36	0.984	1.253	1.118	0.089583	0.17865	0.114191
37	1.05	1.291	1.093	0.094219	0.176798	0.113676
38	1.233	1.316	1.206	0.093423	0.171136	0.114616
39	1.269	1.263	1.316	0.087878	0.17443	0.111128
40	1.295	1.173	1.313	0.084538	0.163673	0.111249
41	1.294	1.24	1.22	0.082924	0.16357	0.115374
42	1.266	1.07	1.226	0.088674	0.162695	0.11595
43	1.269	1.194	1.191	0.118103	0.173401	0.113433
44	1.176	1.197	1.277	0.092605	0.173658	0.113918
45	1.196	1.138	1.348	0.12667	0.167224	0.115192
46	1.208	1.178	1.176	0.165462	0.165063	0.111522
47	1.274	1.28	1.247	0.172234	0.164548	0.111067
48	1.138	1.159	1.084	0.202868	0.153842	0.127021
49	1.12	1.11	1.206	0.263749	0.154563	0.124473
50	1.171	1.123	1.222	0.280952	0.159092	0.122047
51	1.065	1.088	1.212	0.186869	0.152761	0.115344
52	1.172	1.063	1.335	0.128238	0.159658	0.114495
53	0.987	1.103	1.096	0.110944	0.162901	0.115071
54	0.897	1.193	1.158	0.112285	0.149828	0.119651
55	1.015	1.038	0.951	0.079925	0.146534	0.115586
56	0.982	1.043	1.081	0.072721	0.145761	0.107458
57	0.965	1.139	1.135	0.073448	0.149673	0.101999
58	0.997	1.141	1.065	0.073152	0.143034	0.104
59	0.953	1.024	1.026	0.072357	0.144063	0.109793
60	0.897	1.19	0.95	0.072675	0.13907	0.101908
