# Supplementary material for: Mitochondrial inner membrane permeabilisation enables mtDNA release during apoptosis
Source: EMBO J. 2018 Jul 26;37(17):e99238. doi: 10.15252/embj.201899238 (PMC6120664; doi:10.15252/embj.201899238)
Supplement: Supplementary file 17 — Source Data for Figure 5 [file EMBJ-37-e99238-s015.pdf]

Fig 5B

Wt	Drp1-/-
70.73	64.28
68.88	66.66
69.56	58.62
	82.85

Fig 5G

Wt	CypD-/-
83.93	92.59
82.76	89.19
81.25	97.22
68	77.14
71.95	70.37

Fig 5E

Omi Release	Calcein Release
0	360
0	420
0	540
0	420
0	420
0	540
0	660
0	1260
0	1300
0	720
0	390
0	690
0	390
0	240
